# Tissue engineering approaches for the construction of a completely autologous tendon substitute

**DOI:** 10.4103/0970-0358.41109

**Published:** 2008

**Authors:** Bassetto Franco, Vindigni Vincenzo, Dalla Vedova Alessandro, Carolin Tonello, Giovanni Abatangelo, Francesco Mazzoleni

**Affiliations:** Clinic of Plastic Surgery, Department of Medical and Surgical Specialities, University of Padova, Padova, Italy; 1 Department of Histology, Microbiology and Medical Biotechnology, University of Padova, Padova, Italy

**Keywords:** Biomaterials, bioreactor, mechanobiology, tendon, tissue engineering

## Abstract

Tissue engineering is a multidisciplinary field that involves the application of the principles and methods of engineering and life sciences towards i) the fundamental understanding of structure-function relationships in normal and pathological mammalian tissues and ii) the development of biological substitutes that restore, maintain or improve tissue function. The goal of tissue engineering is to surpass the limitations of conventional treatments based on organ transplantation and biomaterial implantation. The field of tendon tissue engineering is relatively unexplored due to the difficulty in *in vitro* preservation of tenocyte phenotype. Only recently has mechanobiology allowed us to gain a better understanding of the fundamental role of *in vitro* mechanical stimuli in maintaining the phenotype of tendinous tissue. This review analyzes the techniques used so far for *in vitro* regeneration of tendinous tissue.

## INTRODUCTION

Tendons are soft connective tissues, which connect muscle to bone and form a musculo-tendinous unit, whose primary function is to transmit tensile loads generated by muscles to move and stabilize joints. The biomechanical properties of tendons can be attributed to the highly defined organization of their extracellular matrix (ECM). ECM of tendons is primarily composed of collagen I, and is organized in a hierarchy of bundles that are aligned in a parallel manner in a proteoglycan matrix. Tendon injuries produce considerable morbidity, and the disability that they cause may last for several months despite what is considered appropriate management. The basic cell biology of tendons is still not fully understood, and the management of tendon injury poses a considerable challenge for clinicians. After an injury, the healing process in tendons results in the formation of a fibrotic scar. The structural, organizational, and mechanical properties of this healed tissue are insufficient as tendons possess a limited capacity to regenerate.[1,2]

Adhesion formation after intrasynovial tendon injury poses a major clinical problem. Disruption of the synovial sheath at the time of the injury or surgery allows granulation tissue and fibroblasts from the surrounding tissue to invade the repair site. Exogenous cells predominate over endogenous tenocytes, allowing the surrounding tissue to attach to the repair site, resulting in adhesion formation. Despite remodeling, the biochemical and mechanical properties of healed tendon tissue never match those of intact tendon. In a study of transected ovine Achilles tendons that had spontaneously healed, the rupture force was found to be only 56.7% of the normal force after twelve months. One possible reason for this is the absence of mechanical loading during the period of immobilization.[1,2] It is well demonstrated that mechanical loading plays a central role in tenocyte proliferation and differentiation, and that the absence of mechanical stimuli leads to a leak of cellular phenotype.[1,2] While certain tendons can be repaired by suturing the injured tissue back together, some heal poorly in response to this type of surgery, necessitating the use of grafts.[3] Unfortunately, finding suitable graft material can be problematic. Autografts from the patient may result in donor site morbidity, while allografts from cadavers may cause a harmful response from the immune system besides also being limited in supply. In both cases, the graft often does not match the strength of the undamaged tissue.[4] The loss of mechanical properties is mainly due to a distorted ECM composition and an architectural misalignment of collagen fibrils in the scar tissue.

For this reason, obtaining tendinous tissue through tissue engineering approaches becomes a clinical necessity. Tissue engineering is a multidisciplinary field that involves the application of the principles and methods of engineering and life sciences towards i) the fundamental understanding of structure-function relationships in normal and pathological mammalian tissues and ii) the development of biological substitutes that restore, maintain or improve tissue function. The goal of tissue engineering is to surpass the limitations of conventional treatments based on organ transplantation and biomaterial implantation. It has the potential to produce a supply of immunologically tolerant, ‘artificial’ organs and tissue substitutes that can grow within the patient. This should lead to a permanent solution to the damage caused to the organ or tissue without the need for supplementary therapies, thus making it a cost-effective, long-term treatment. Tissue engineering uses different biomaterials, carrier cells and/or bioactive factors to stimulate tissue regeneration. Nowadays, there are many tissue engineering approaches. The earliest clinical application of human cells in tissue engineering (in 1980) was for skin tissue using fibroblasts, and keratinocytes, on a scaffold. During the last 30 years, many innovative approaches have been proposed to reconstruct different tissues: skin, bone, and cartilage. The field of tendon tissue engineering is relatively unexplored due to the difficulty in *in vitro* preservation of tenocyte phenotype, and only recently has mechanobiology allowed a better understanding of the fundamental role of *in vitro* mechanical stimuli in maintaining the phenotype of tendinous tissues. Tendon tissue engineering requires a scaffold that functions as a temporary structure to support initial tissue growth. Scaffolds can improve tendogenesis allowing cell proliferation, ECM production and finally, organizing the matrix into functional tendon tissue. Moreover, tendon regeneration could be stimulated through approaches such as the use of growth factors and gene therapy, as well as by *in vitro* mechanical forces. This review analyzes the techniques used so far for the *in vitro* regeneration of tendinous tissues, and discusses strategies for the improvement of the same.

### Scaffold materials used in tendon tissue engineering

Recreating a scaffold appropriate for tendon tissue engineering is difficult because of the particular micro-architecture, both of collagen fibres and of ECM molecules. It is clear that the resistance and elasticity of tissues depend on these parameters due to which, competent scaffolding materials are needed. Biomaterials should protect the cells and new tissue from strong forces, while allowing graded exposure to loading at later points in time. This will allow the tissue to develop more naturally and function more efficiently.[5] There are many scaffold materials, both natural and synthetic. Natural scaffolds are composed of collagen,[6,7] porcine small intestine submucosa,[8,9] chitosan-based scaffolds,[10–12] silk fibers,[13–15] semitendinosus tendon[16] and fibronectin/fibrinogen fibres.[17] In addition to these natural scaffolds, there are a large number of synthetic scaffolds developed for tendon tissue engineering. The most commonly used synthetic scaffolds are made of poly-lactic acid (PLA)[11,18–20] and poly lactic-coglycolic acid (PLGA).[18,21,22]

Usually, a 3D scaffold housing a specific cell type has to be able to be directed to form tendon/ligament tissue. As compared to 2D culture, 3D culture offers the advantage of more closely recreating the spatial organization of native tissue.[23] There are many considerations that should be taken into account when engineering tendon or ligament tissues. The scaffold should encourage cellular recruitment and tissue ingrowth. Early in the repair process, the scaffold should maintain its mechanical and architectural properties to protect cells and the new, growing tissue from strong forces and early inflammatory events. Subsequently, the scaffold should be gradually reabsorbed allowing a controlled exposure of the regenerating tissue to the local cellular, biochemical and mechanical environment. This will allow the tissue to develop more naturally and function more efficiently. In order to avoid stress shielding, the scaffold should ideally degrade at the same rate that the new tissue is created. In order to ensure final clinical use, neither the scaffold nor its degradation products should be harmful to the surrounding tissue and they should not result in unresolved inflammation or other deleterious biological responses. In response to these stringent and varied criteria, a number of scaffold materials, both natural and synthetic, have been examined. Collagen, the most prevalent structural protein in the human body, is a natural scaffold material for ligament and tendon replacement. Cells cultured in collagen gels produce extracellular matrix and align longitudinally with the long axis of the tissue equivalent, thereby mimicking cell alignment in ligaments *in vivo*.[4,24] Fibroblasts seeded in collagen gels change their shape and orientation over time[24–27] and these organizational changes have been correlated with cell proliferation, protein synthesis, and matrix morphogenesis.[28–30] Fibroblast-seeded collagen scaffolds have been investigated with regard to their ability to accommodate cell attachment, proliferation, and differentiation.[4,24,31,32] An *in vivo* study showed that fibroblast-seeded collagen scaffolds were viable for at least eight weeks after reimplantation into donor rabbits.[33,34] In another study, a tissue-engineered, ligament-like structure was derived from Anterior Cruciate Ligament (ACL) fibroblasts seeded on a collagen scaffold that was anchored to bone, to facilitate implantation.[4] Elongation of the structure by tension induced a parallel orientation of type I collagen fibers, and this organization was progressively modulated by the fibroblasts seeded into the structure and by the *in vitro* application of tension during its development. Knitted Dacron scaffolds seeded with canine fibroblast cells demonstrated a more uniform and abundant encapsulation with connective tissue than unseeded scaffolds.[33] These encouraging cellular results lay the foundation for the increasingly challenging goal of improving the *in vivo* mechanical strength to the level of functional ligaments or tendons. Fibres of collagen or degradable polymers of PLGA and PLA can be crosslinked, woven, or braided to increase the mechanical strength of the resulting tissue-engineered tendons and ligaments.[32,34–40] Fibroblasts have been shown to attach to and function on collagen fibres or PLGA scaffolds in *in vitro* studies, and fibroblast-seeded collagen fiber scaffolds have shown promising results in implantation studies.[31,32,41] Silk protein-based matrices have been investigated for ACL tissue engineering because of their interesting mechanical properties as well as their biocompatibility and biodegradability.[42,43] A silk fibre matrix has promising mechanical properties (tensile strength, stiffness, yield point, and elongation to failure) comparable to those of human ACL.[43] *In vitro* data demonstrate that the silk matrix provides sufficient support for the attachment, proliferation, and differentiation of human Mesenchymal Stem Cells (MSCs). The fibrous scaffold was covered with a uniform cell sheet and cell-associated extracellular matrix after 21 days with few changes in the tensile strength of the fibre matrix. In an *in vivo* approach, a surgically created gap Medial collateral ligament (MCL) injury in rabbits was healed by using porcine small intestinal submucosa as a collagen scaffold.[44]

### Cells

Multiple cell types have been seeded within scaffolds in an effort to stimulate cell-mediated tissue regeneration. The most common cell types employed are fibroblasts, tenocytes and mesenchymal stem cells/marrow stromal cells (MSCs).[5] The main cell type found in tendon tissue is the fibroblast, which is responsible for secreting and maintaining the extracellular matrix. Hence, fibroblasts are the predominant cell type used for tissue engineering applications.[5] Two different fibroblast populations can be found in the tendon: the elongated tenocytes and the ovoid-shaped tenoblasts.[45] Elongated tenocytes proliferate well in culture and have optimal morphology in terms of expression of collagen type 1, which is a major component of normal tendons.[45] Tendon cells are usually isolated from human tendon samples by tissue dissociation techniques.[46–48] After two or three cell culture passages and before they lose their phenotype, they are seeded into collagen gels or into scaffolds at an appropriate cell density (10^6^ cells/mL).[46–48] Several *in vivo* and *in vitro* studies have showed the role of MSCs obtained from different human tissues (mainly bone marrow and adipose tissues) in tendon engineering.[46,49,50] MSCs can be stimulated to differentiate into fibroblasts when exposed to mechanical stress,[51] and their rates of proliferation and collagen excretion have been shown to be higher than those of fibroblasts, so they may be a viable alternative to fibroblasts.[45]

Kall and colleagues[52] from Hanover Medical School, investigated techniques using mesenchymal stem cells for *in vitro* tendon engineering using a collagen type I gel influenced by cyclic stretching. Tendon substitutes were created by dispersing human mesenchymal stem cells in a collagen type I gel, followed by polymerization in glass cylinders with defined measurements.[52] A bioreactor was fabricated to expose tissue engineered tendons to cyclic loading for three weeks. This was a fascinating study and demonstrated histologically that the stretched constructs showed longitudinally oriented spindle-shaped cells with an organized matrix and parallel collagen fibres and increased mRNA syntheses of collagen type I, type III, and fibronectin.[52] Biomechanical studies will demonstrate whether these constructs have any clinical application. Kryger *et al.*[53] compared tenocytes and mesenchymal stem cells for use in flexor tendon tissue engineering. They studied four candidate cell types for use in reseeding acellularised tendon constructs. Specifically, they compared epitenon tenocytes, tendon sheath fibroblasts, bone marrow-derived mesenchymal stem cells (BMSCs), and adipoderived mesenchymal stem cells (ASCs) with respect to their *in vitro* growth characteristics, senescence and collagen production, as well as the viability of reseeded constructs.[53] They also studied the *in vitro* viability of tendon constructs after reseeding and after *in vivo* implantation in a clinically relevant model of rabbit flexor tendon grafting. Results showed that epitenon tenocytes, tendon sheath cells, bone marrow and adipo-derived stem cells have similar growth characteristics and can be used to successfully reseed acellularized tendon grafts.[53] Constructs using the four cell types were also successfully implanted *in vivo* and showed viability after six weeks following implantation.[53] The most relevant novel finding is that adipo-derived mesenchymal stem cells showed higher proliferation rates at later passages when compared with epitenon tenocytes. ASCs have been shown to have multipotency and may be driven toward tenocyte differentiation when seeded into tendon constructs and exposed to the appropriate environment and mechanical forces.[53] As confirmed by immunocytochemistry analysis, these stem cells also produce collagen, suggesting that they would contribute to *in vivo* tendon matrix remodeling. In conclusion, these results suggest that ASCs have a practical advantage when compared with epitenon tenocytes and sheath fibroblasts, given that it is easier to harvest large amounts of fat tissue.

### Local delivery of growth factors

*In vitro* cell proliferation and differentiation require an intake of ions and nutrients from the culture medium. However, to obtain specific phenotype expression and proper cell differentiation, several biochemical factors such as cytokines and growth factors, must be added to the culture medium.[46,54] The main growth factors that affect the growth and differentiation of ligament and tendon tissues include fibroblast growth factor (FGF), platelet-derived growth factor-BB (PDGF-BB), epidermal growth factor (EGF), insulin-like growth factor (IGF)-1 and members of the transforming growth factor-β (TGF-β)/bone morphogenetic proteins (BMPs) family.[46,55,56] Many of these growth factors are physiologically released at the site of injury and are able to stimulate cell proliferation, migration, differentiation and matrix synthesis.[55] Direct injection of recombinant FGF into injured rat patellar tendons increases cell proliferation and Type III collagen expression.[55,56] Injection of PDGF-BB has been shown to increase the mechanical properties of the healing ligament.[55,57,58] To overcome a potential overload of growth factors after local administration, time-dependent administration of PDGF-BB was investigated recently for tendon healing.[55,59] It has also been shown that EGF, IGF-1 and PDGF-BB individually stimulate fibroblast proliferation. IGF-1 seems to possess anti-inflammatory functions and also acts as a chemotactic attractant for endothelial cells.[55,60] TGF-β1 was originally shown to be involved in chick distal limb tendon formation during embryonic development. After local administration, TGF-β stimulates collagen synthesis and in combination with EGF, stimulates an improved healing of ligaments.[55,61] There is now ample evidence that growth and differentiation factors 5, 6 and 7 (GDF 5, 6, 7); also termed cartilage-derived morphogenetic proteins 1, 2, 3 [CDMP 1, 2, 3] or BMP 14, 13, 12, respectively and all members of the BMP family of growth factors, affect tendon and ligament formation.[55] Inactivation of GDF 5 and GDF 6 genes in mice causes defects in ligaments.[55,62–64] A role for GDF 5, 6 or 7 in tendon or ligament formation has also been demonstrated in that, tendon-like structures result after the ectopic implantation of these factors into subcutaneous or intramuscular sites in adult rats.[55,65] GDF 5, 6 and 7 were also used successfully in dose-response studies for rat Achilles tendon healing; GDF 7 (BMP 12)-transfected pluripotent mesenchymal stem cells contributed to the healing of a tendon defect.[55,66–68] The role of TGF-β/BMP family members in tendon/ligament formations was substantiated in a recent study showing that a specific signalling mediator of the TGF-β/BMP family, Smad 8, has tenogenic potential.[55,69] In conclusion, growth factors from various origins and sources have the capacity to promote tendon healing. However, an understanding of the right time for the administration of growth factors and their dosage is a prerequisite to design effective growth factor therapy.

### Gene therapy

Gene therapy delivers genetic material (DNA) to cells, allowing the modification of cellular function by means of viral or nonviral vectors or direct gene transfer.[70] Gene therapy enables the delivery of individual proteins to specific tissues and cells.[70–73] Several animal studies have been conducted to investigate the feasibility of gene transfer to tendons.[70,74] For example, hemagglutinating virus of Japan (HVJ)-liposome constructs were used to deliver β-galactosidase to rat patellar tendons. *In vivo* and *ex vivo* gene transfer techniques have been used as well, as a result of which, sustained gene expression seems to last for about six weeks, possibly long enough for clinical applications.[70,75,76,77] *Ex vivo* gene transduction is possibly more efficient, but the techniques must be optimized. Gene therapy can also alter the healing environment of tendons in animal models of tendon repair. Adenoviral transduction of focal adhesion kinase (FAK) into partially lacerated chicken flexor tendons resulted in an expected increase in adhesion formation and a twofold increase in the work required for flexion compared with the results in control groups.[70,78] These differences were significant (*P* = 0.001). While tendon healing was not improved in this study, the results did demonstrate that the healing environment and conditions could be manipulated.[70,78] Bone morphogenetic protein-12 (BMP-12), the human analog of murine GDF-7257, is found to increase the expression of procollagen types I and III genes in human patellar tenocytes, and it is found at sites of tendon remodelling.[70,79,80] BMP-12 increases the synthesis of type I collagen by 30% in chicken flexor tenocytes, and the application of tenocytes transfected with the BMP-12 gene to a chicken flexor tendon laceration model resulted in a twofold increase in tensile strength and load to failure after four weeks.[70,81] Transfer of genes to tendons is feasible, and as the healing environment can be manipulated for up to eight to ten weeks, this may be long enough to be clinically relevant. The above studies were conducted in tendon transfection models; in addition to this, the delivery of substances such as platelet-derived growth factor-B (PDGF-B), BMP-12, and decorin may improve healing of tendinopathy; however, additional research is required.[70,81–88]

### Organizing cells within the scaffold - Tendon engineering by the application of mechanical load

Studies in developmental biology and wound healing have revealed many of the biological events and signals involved in tendon cells and tissue morphogenesis.[88–90] It is important for tissue engineers to understand these events as it may be necessary to employ the principles of developmental biology in designing the appropriate microenvironment for tissue regeneration. *In vitro* tissue development may include the application of mechanical loading to precondition the engineered tissue for the *in vivo* mechanical environment. Mechanical stress plays a significant role in modulating cell behavior and has driven the development of mechanical bioreactors for tissue engineering applications.[89–92] Tendons transmit force from the muscle to the bone and act as a buffer by absorbing external forces to limit muscle damage. Tendons exhibit high mechanical strength, good flexibility, and an optimal level of elasticity to perform their unique role. Tendons are visco-elastic tissues that display stress relaxation and creep. The mechanical behavior of the constituent collagen depends on the number and types of intramolecular and intermolecular bonds.

**Figure 1:**
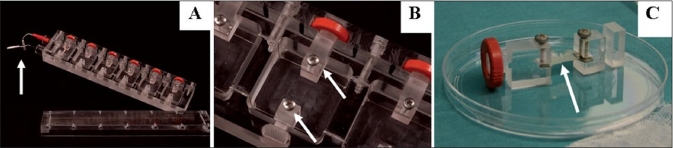
Bioreactor. We developed a bioreactor able to reproduce a cycle mechanical stretching onto cell-seeded biomaterials (A). White arrow in (A) points at the electrical connection, in (B) shows a detail of biomaterial anchoring system and in (C) points at stretched biomaterial

**Figure 2:**
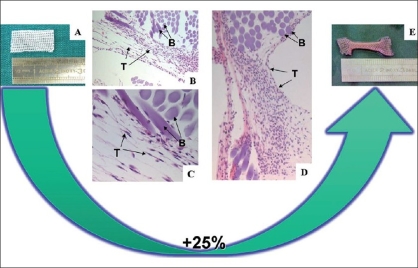
Diagram of our *in vitro* experiment. (A) Biomaterial before stretching; (B and C) tenocytes (T) seeded onto biomaterial (B) after 6 days of culture under mechanical cyclic tension; (D-E) cell-biomaterial culture system after 12 days: mechanical stress is a factor that is thought to play an essential role in tissue generation and reparation processes. We have observed that cyclic stretching of cells induces various biological responses, including cell proliferation and longitudinal alignment

Experiments have confirmed cell growth and function would be controlled locally through physical distortion of the associated cells or through changes in cytoskeletal tension. Moreover, experimental studies have demonstrated that cultured cells can be switched between different fates including growth, differentiation, apoptosis, directional motility or different stem cell lineages, by modulating cell shape.[90–93] Kessler *et al.*[46,94] demonstrated that collagen fibres and tendon cells can be oriented along the direction of the stress and can upregulate synthesis of tissue inhibitor matrix metalloproteinases-1 and −3 as well as of collagen type I, the main component of tendinous extracellular matrix. Moreover, *in vitro* cyclic strain allows an increased production of TGF-β, FGF and PDGF by human tendon fibroblasts.[46,95] Cyclic stretching of collagen type I matrix seeded with MSCs for 14 days (8 h/day) resulted in the formation of a tendon-like matrix.[46,96] Expression of collagen types I and III, fibrinonectin and elastin genes was found to have increased when compared with nonstretched controls in which no ligament matrix was found.[46,97] We have observed that nonwoven hyaluronic acid biomaterial allows the development of 3D cultures, which allows tenocyte integration and proliferation. Mechanical traction enhances cell proliferation and their longitudinal alignment [Figures 1 and 2]. The model reproduces *in vivo* tendon healing by preventing differentiation of tenocytes into fibroblasts. Other experiments have demonstrated the beneficial effects of motion and mechanical loading on tenocyte function.[70,98] Repetitive motion increases DNA content and protein synthesis in human tenocytes in culture.[70,98] Even fifteen minutes of cyclic biaxial mechanical strain applied to human tenocytes, results in improved cellular proliferation.[70,99]

In animal experiments, mechanical stretching has improved the tensile strength, elastic stiffness, weight and cross-sectional area of tendons.[70,100,101] These effects result from an increase in collagen and extracellular matrix network syntheses by tenocytes. Application of a cyclic load to wounded avian flexor tendons results in the migration of epitendon cells into the wound.[70,102] In rabbit patellar tendons, application of a 4% strain provides protection against degradation of mechanical stretching by bacterial collagenase.[70,103]

Clinical studies have shown the benefit of early mobilization following tendon repair, and several postoperative mobilization protocols have been advocated.[70,104–108] The precise mechanism by which cells respond to load remains to be elucidated.[70] However, cells must respond to mechanical and chemical signals in a coordinated fashion. For example, intercellular communication by means of gap junctions is necessary to mount mitogenic and matrigenic responses in *ex vivo* models.[70] Duration, frequencies and amplitude of loading directly influence cellular response and behavior in many other tissues. Understanding the physiological window for these parameters is critical and represents future challenges of research in tendon tissue engineering.

## CONCLUSIONS

Technological improvements in the field of tissue engineering are leading to new potential developments in the approaches being used to treat tendon injuries. An integration of mesenchymal stem cells, growth factors, mechanical stimuli (bioreactor) and bioresorbable polymers can provide a solution for the treatment of difficult tendon injuries.

Despite preliminary *in vitro* and *in vivo* results, several objectives still remain unaccomplished for complete tendon regeneration: i) there is no scaffold able to simultaneously respond to major requirements like biocompatibility, biofunctionality, mechanical properties and processability; ii) cell culture procedures to be performed on scaffolds are not yet satisfactory, often resulting in a low rate of cellular adhesion and of ECM deposition; iii), there is currently a significant gap between *in vitro* results and *in vivo* application of tissue-engineered tendon tissue. Moreover, our knowledge is still limited in the field of growth factor and gene therapy for tendon regeneration, being based only on empirical observations rather than on a thorough understanding of the underlying mechanisms and pathways.[109] Future research is needed to show that the extracellular matrix produced *in vivo* in response to the cell/growth factor/polymer composites, is effective and functional as a regenerated tissue.
